# The Impact of Dimension Switching on Visual Short-Term Memory

**DOI:** 10.1177/17470218251404415

**Published:** 2025-11-28

**Authors:** Stuart B. Moore, James A. Grange

**Affiliations:** 1 School of Psychology, Keele University, UK

**Keywords:** vSTM, task switching

## Abstract

Visual short-term memory (vSTM) refers to the subset of the cognitive system responsible for storing visual information over short periods of time. While much research has focused on its capacity limitations, less is known about how vSTM operates in dynamic environments where priorities shift across feature dimensions. In this study, we bridge research on vSTM and cognitive control by embedding change detection (Experiments 1 and 2) and delayed estimation (Experiment 3) paradigms within a task switching paradigm, where the relevant feature dimension (colour or orientation) either repeated or switched across trials. Across all experiments, we observed a cost to vSTM performance on switch relative to repetition trials. Mixture modelling of delayed estimation responses revealed that these switch costs were not due to reduced memory precision or memory failures, but rather to a selective increase in non-target responses reflecting feature–location binding errors. We propose that dimension switching selectively impairs the binding of feature values to locations and allows interference from irrelevant (but recently attended) feature dimensions. Our findings demonstrate that vSTM is sensitive to failures of attentional control, not just capacity limits.

## Introduction

Visual short-term memory (vSTM) refers to the subset of the cognitive system responsible for the retention of visual information over short periods of time (Phillips, 1974). Considerable work has been directed at understanding the nature of vSTM, in particular, understanding its capacity limitations (for reviews, see Bays et al., 2024; Ma et al., 2014). Such an understanding is essential as vSTM is the interface between sensory perception of the external visual world and higher-order cognition. Understanding these capacity limitations, therefore, not only provides insight into fundamental constraints of cognitive processing but it also informs how vSTM interacts with more complex cognitive functions.

A consensus emerging in the literature is that capacity limitations arise in vSTM due to the allocation of a limited, continuous, memory resource across memoranda (Bays & Husain, 2008; Bays et al., 2009, 2024; van den Berg et al., 2012; for an alternative view see Luck & Vogel, 2013; Zhang & Luck, 2008). When the number of visual elements to retain is low, each element receives plentiful resources and is encoded and retained in vSTM with high fidelity, leading to enhanced probability and precision of later recall. Conversely, when the number of elements to retain is high, each element receives considerably fewer resources, resulting in reduced probability and precision of later recall. Capacity limitations arise—and memories therefore fail—when memoranda are not represented with sufficient precision due to insufficient allocation of limited resources (Bays et al., 2024).

Understanding how memoranda are represented in vSTM is essential for understanding vSTM capacity limitations because it determines at what level the limited memory resources are applied. Whether vSTM representation is object-based (i.e. memory resources are applied to whole objects, D. Lee & Chun, 2001; Luck & Vogel, 1997; Ngiam et al., 2024; Ramaty & Luria, 2025; Sone et al., 2021; Vogel et al., 2001) or feature-based (i.e. memory resources are applied to individual features such as colour or orientation, Bays et al., 2011; Brady et al., 2011; Brown et al., 2021; Fougnie et al., 2010; Li et al., 2022; Markov et al., 2019, 2021; Marshall & Bays, 2013; Shin & Ma, 2016, 2017) has been a topic of intense debate, with recent reviews generally favouring a feature-based account (Bays et al., 2024; Brady et al., 2024).

Early research into vSTM representation used the change detection paradigm in which participants are briefly (e.g. 100 milliseconds) presented with an array of objects of varying set size to encode. After a retention interval (e.g. 1,000 ms) in which the objects are removed, participants are presented with a second array of objects in which one object may have changed (e.g. from red to black). It is the participants’ task to make a change/no-change judgement on the test array. Whilst early research suggested change detection performance is unaffected by the number of feature dimensions per object (Luck & Vogel, 1997; but see Wheeler & Treisman, 2002)—consistent with models assuming object-based representation in vSTM—later research has shown that the precision of memory representations does decrease with increasing features (Fougnie et al., 2010). In addition, it has been shown that memory capacity is more readily consumed by complex, multi-feature, objects in comparison to simple objects (Alvarez & Cavanagh, 2004), which is hard to reconcile with a purely object-based representation in vSTM.

Further evidence for feature-based representation in vSTM comes from studies that show memory performance for each feature dimension of a single object is independent. This independence is evidenced using the delayed-estimation paradigm (Prinzmetal et al., 1998; Wilken & Ma, 2004). This paradigm is similar to the change detection paradigm in that participants encode a set of memoranda, followed by a retention interval. However, at test, participants are cued to the location of one of the memoranda and must recall its feature value by reproducing it on a continuous scale. For example, in a task requiring memory for colour, participants are presented with a colour wheel at test and select the colour that best matches the recalled feature value of the cued item. Memory precision is then estimated by taking the angular difference between the true target feature value and the participant’s response. Supporting the feature-based representation account, Fougnie and Alvarez (2011) found that when participants were asked to remember objects with colour and orientation features, memory precision for an object’s colour remained high even when precision for that object’s orientation was very low (and vice versa), supporting the view that feature dimensions are represented independently (see also Bays et al., 2011; but see Ramaty & Luria, 2025).

### The Current Study

The need to allocate a limited memory resource across multiple features distributed across multiple objects presents an interesting challenge for the cognitive system: Since the resource is limited—and since successful (and precise) recall of visual information requires that memoranda receive sufficient resources—it is imperative for the cognitive system to prioritise the allocation of this limited resource to features that are task-relevant if goal-directed behaviour is to be achieved. As the limited vSTM resource is thought to be flexible in that it can be concentrated more on certain features to enhance their representation (Bays & Husain, 2008), cognitive control is required in dynamic environments when goals change to ensure that memory resources are allocated optimally to task-relevant features. Without such control, task-irrelevant features compete for a share of limited vSTM resources, which in turn reduces the precision of all memoranda.

This challenge is similar to the one faced by the cognitive system during task switching. In typical task-switching paradigms, participants are presented with stimuli that afford multiple tasks (e.g. digits) and are cued on each trial to perform one of two tasks. For example, participants may be presented with a cue “Parity” which requires them to judge whether the digit is odd or even, or the cue “Magnitude” which requires them to judge whether the digit is lower or higher than five. The critical manipulation is whether the task on the current trial has switched from that of the previous trial (e.g. Parity to Magnitude) or whether it has repeated (e.g. Magnitude to Magnitude). It is a consistent finding that response time and errors are increased on task switch trials relative to task repetition trials (Grange & Houghton, 2014; Kiesel et al., 2010; Koch et al., 2018; Logan, 2003; Monsell, 2003; Vandierendonck et al., 2010).

Task performance requires establishing a stable mental representation—a so-called *task set*—that comprise attention settings (*stimulus sets*, i.e. which object/feature to attend to) and stimulus-response mappings (*response sets*, i.e. how to respond to these objects) necessary to perform a particular task (Grange, 2025; Schneider, 2014; Schneider & Logan, 2007). The switch cost in task switching performance has been explained by (a) time-consuming reconfiguration of the relevant task set component on switch trials that is not required on repetition trials (Monsell, 2003; Rogers & Monsell, 1995), (b) interference caused by persisting activation of the previously relevant task set component on switch trials (i.e. carryover effects, Allport et al., 1994), and (c) a combination of both reconfiguration and interference (Meiran, 2000; Meiran et al., 2000; Vandierendonck et al., 2010). Recent work has shown that stimulus set and response set are unique components of the overall task set that can be independently controlled and updated, and each contributes to the overall switch cost (Grange, 2025).

The current study examines how the allocation of limited memory resources to task-relevant features in vSTM is influenced by changes in task demands in dynamic environments. Specifically, we embed vSTM change detection (Experiments 1 and 2) and delayed-estimation (Experiment 3) paradigms within a task switching paradigm where the relevant feature dimension on each trial either repeats (e.g. colour–colour) or switches (e.g. colour–orientation) from that of the previous trial. In conventional vSTM tasks, which hold the relevant feature dimension constant, participants can adopt a stable strategy by maintaining consistent allocation of resources; in contrast, the paradigm reported in the current work requires participants to dynamically adjust which feature is prioritised on a trial-by-trial basis, helping elucidate the interplay between dynamic task demands and memory resource allocation. We expect that—analogous to classic task switching—there will be a cognitive cost when the target feature changes.

## Experiments 1a and 1b

In Experiment 1, a change detection paradigm was used to explore the impact of dimension switching on vSTM performance. In Experiment 1a, bivalent stimuli (coloured circles with an orientation) were used, whereas in Experiment 1b, univalent stimuli (two coloured circles with no orientation, and two circles with an orientation but no colour) were used. The total set size in each Experiment was four, although the effective set size (i.e. the set size of stimuli with features of the relevant dimension) in Experiment 1b was two. The feature load was eight and four in Experiments 1a and 1b, respectively.

### Method

#### Participants

A total of 57 participants completed Experiment 1a and 45 completed Experiment 1b. As these experiments were part of the initial exploratory set, our sample size was determined by collecting data from as many participants as possible within a specified time frame. Participants whose proportion accuracy was not significantly better than chance (i.e. 0.50, as determined by a binomial test) were removed. As a result, the final sample size was 44 for Experiment 1a and 45 for Experiment 1b. Participants were recruited via Prolific Academic (prolific.co), and both experiments had unique participants. Participants were aged between 18 and 60 years of age (inclusive)^1^ and self-reported normal or corrected-to-normal visual acuity and colour vision. Recruitment was limited to participants from the United Kingdom and the United States. Participants were paid a small fee for participation. A constraint was added to allow only those accessing the experiment from a desktop or laptop computer.

#### Materials

**Figure 1.**
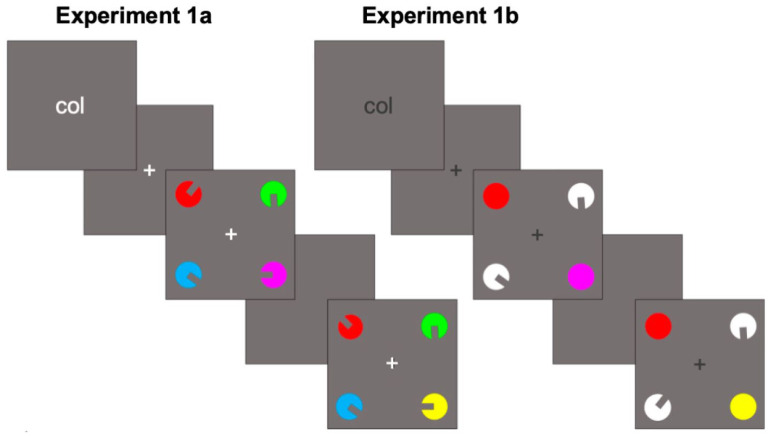
An example trial from Experiment 1a (left) and Experiment 1b (right). In both examples, the cue “col” indicates participants must memorise the colour of the stimuli on the first screen (the memory display). After a short retention interval, the test display appears, and participants must judge whether a stimulus has changed on the cued feature dimension. In both examples, the bottom-right stimulus has changed feature value. Note that in all trials, a change always occurs in the uncued feature dimension (e.g. the orientation of the top-left stimulus in example A and the bottom-left stimulus in example B).

The experiment was programmed and delivered using Gorilla (Anwyl-Irvine et al., 2020). Stimuli in Experiment 1a consisted of four bivalent circular shapes with a rectangular segment removed to indicate orientation (i.e. each stimulus had two feature dimensions of colour and orientation). In Experiment 1b, all stimuli were univalent with a single feature dimension: two stimuli were coloured circles (with no segment cut out and thus no orientation), and two stimuli were white circles with an orientation (see Figure 1, e.g. stimuli from each Experiment).

The feature dimension of colour could take on one of seven colours in both experiments (defined here with the Red, Green, Blue [RGB] values): Black (0, 11, 16), blue (65, 105, 225), cyan (20, 253, 255), green (0, 250, 3), purple (255, 41, 255), red (255, 54, 31), or yellow (253, 254, 21). The univalent orientation stimuli in Experiment 1b were presented in white (255, 255, 255), which was not a possible colour feature dimension. The feature dimension of orientation could be any value between one and 360 degrees with the constraint that the minimum orientation difference between all stimuli on a single trial was 40 degrees. The RGB value for the background was light grey (127, 128, 128). Stimuli were presented within a dark grey (89, 89, 89) frame positioned in the centre of the display. Feature dimension cues on each trial were either the word “col” (to cue attention to the colour feature dimension) or “ori” (to cue attention to the orientation feature dimension). In Experiment 1a, dimension cues and fixation crosses were white, but were dark grey in Experiment 1b due to the use of white stimuli.

#### Procedure

In both experiments, a change detection paradigm was used. Participants were required to monitor for a change in any of the stimuli across two successive screens (the memory display and the test display). Importantly, on each trial, participants were presented with a cue that indicated which feature dimension—colour or orientation—should be monitored. Participants completed two types of blocks across the experiment: In *pure blocks*, the relevant feature dimension repeated throughout the whole block (i.e. participants only monitored for changes in colour *or* orientation). In *mixed blocks*, the relevant feature dimension was randomly selected on each trial such that on some trials the relevant feature dimension could repeat from that of the previous trial (e.g. colour–colour) or switch (e.g. colour–orientation).

Prior to the main experimental blocks, participants completed practice blocks consisting of 40 trials (10 pure colour, 10 pure orientation, and 20 mixed) to familiarise them with the task. Accuracy feedback was provided at the end of each trial in the practice blocks. The main experimental section consisted of a total of 450 trials; these were separated into three cycles following the same pattern of 25 pure orientation trials, 25 pure colour trials, and two blocks of 50 mixed trials. Self-paced breaks were provided every 50 trials.

On each trial, participants were presented with a cue for 500 milliseconds (ms), indicating which feature dimension was relevant for that trial. The cue was then replaced with a central fixation cross for 500 ms, after which the memory display consisting of four stimuli was presented. The stimuli were organised such that each stimulus occupied the corner of an invisible square (see Figure 1). In Experiment 1b, stimuli containing the same feature dimension were constrained to always appear diagonally opposite each other to prevent stimuli for the relevant feature dimension from being presented on one side of the display. The memory display was present for 200 ms, after which time they disappeared and were replaced by a blank screen for a retention interval of 1,500 ms. After this time, the test display was presented for 200 ms. On change trials, the value of one stimulus (randomly selected) on the relevant feature dimension would change, whereas on no-change trials, all values of the relevant feature dimension were identical to the memory display. On all trials, the value of one stimulus (randomly selected) on the irrelevant feature dimension would change.^2^ Participants were instructed to ignore this change and to only monitor for change on the cued feature dimension. On change trials, the stimulus selected to feature the change on the relevant dimension was always different to the stimulus selected to feature the change on the irrelevant dimension.

After the test display had been presented for 200 ms, the stimuli disappeared, and participants were required to make their response: they were instructed to press the “M” key on the keyboard to indicate a change response, and the “Z” key to indicate a no-change response. Participants were instructed to respond as quickly and as accurately as possible. Response time (ms) and accuracy were recorded on each trial. After a response had been registered, the frame went blank for 250 ms, after which a fixation cross appeared for 750 ms before the cue for the next trial was presented.

#### Design

To summarise the design, both experiments featured one independent variable of *Sequence* with three levels (pure repetition vs. mixed repetition vs. mixed switch). We analysed the impact of this independent variable on four dependent variables: proportion accuracy and response time (in milliseconds, ms) were supplemented by measures of signal detection theory d’ and response bias, c (criterion). Following Macmillan and Creelman (2004), signal detection measures were calculated as follows:

(1) $$d^{\prime }=z\left(h\right)-z\left(f\right)$$

and

(2) $$c=-\frac{1}{2}[z\left(h\right)+z\left(f\right)]$$

where z(h) and z(f) are *z*-scored hit rate and false-alarm rate, respectively. Within the context of change detection, hit rate represents the proportion of trials participants correctly identified a change, and false-alarm rate represents the proportion of trials participants responded change when no change was present. c is a measure of response bias; values above zero indicate a conservative bias, where the participant is more likely to respond “no-change.” Values below zero indicate a liberal bias, where the participant is more likely to respond “change.”

### Results

All of the data wrangling, statistical modelling, and visualisation utilised R (R Core Team, 2023) and various packages.^3^ Prior to all analyses, the first trial of each block was removed as these cannot be classified as repetition or switch trials. For accuracy and signal detection analysis, trials immediately following an error were removed. For response time (RT) analysis, error trials and the trials following an error were removed. Correct RTs were also trimmed using the R package *trimr* (Grange, 2022); RTs shorter than 150 ms were removed, as were RTs longer than 2.5 standard deviations above the mean per participant per cell of the design. Bayesian one-way ANOVAs (with the factor dimension *Sequence*, with levels *pure*, dimension-*repetition*, and dimension-*switch*), were conducted for each of the dependent variables proportion accuracy and RT, as well as signal detection theory measures sensitivity (*d*’) and response bias ( c ). If the Bayesian one-way ANOVA showed moderate evidence in support of the presence of an effect (i.e. a $BF_{10}$ greater than 3), Bayesian paired-samples *t*-tests were conducted to quantify the mixing cost (i.e. pure vs. repetition) and switch cost (i.e. repetition vs. switch). All Bayesian analyses were conducted using the *BayesFactor* R package (Morey & Rouder, 2024) using default priors. Interpretation of Bayes factors (BFs) for all analyses utilised the criteria presented by M. D. Lee and Wagenmakers (2014).

#### Experiment 1a

**Figure 2.**
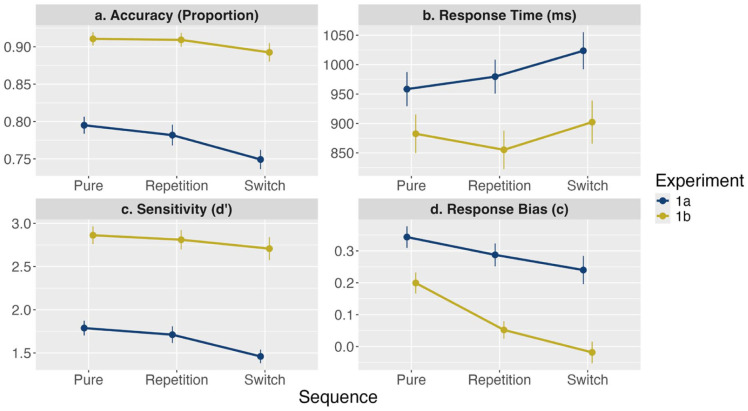
The results from Experiments 1a and 1b. Data points represent the mean for each dependent variable (a) Accuracy, (b) Response Time, (c) Sensitivity, and (d) Response Bias.

All of the results for Experiment 1a and 1b can be seen in Figure 2. Analysis of proportion accuracy (Figure 2a) across Sequence showed extreme evidence for the alternative ( $BF_{10}$ = 1,816.37); follow-up tests showed no evidence for a mixing cost ( $BF_{10}$ = 0.49; Cohen’s d = 0.15, 95% CI [−0.04, 0.35]) but very strong evidence for a switch cost ( $BF_{10}$ = 51.22; d = 0.37 [0.16, 0.57]). Analysis of RT (Figure 2b) across Sequence showed very strong evidence for the alternative ( $BF_{10}$ = 49.65); whilst there was no evidence for a mixing cost ( $BF_{10}$ = 0.38; d = 0.11 [−0.05, 0.27]), there was strong evidence for a switch cost ( $BF_{10}$ = 24.46; d = 0.21 [0.09, 0.34])). For the signal detection analysis, analysis of sensitivity (Figure 2c) across Sequence showed extreme evidence for the alternative ( $BF_{10}$ = 3,234.43); whilst there was no evidence for a mixing cost ( $BF_{10}$ = 0.35; d = 0.12 [−0.07, 0.32])), there was very strong evidence for a switch cost ( $BF_{10}$ = 46.61; d = 0.42 [0.18, 0.66])). Analysis of response bias (Figure 2d) across Sequence showed moderate evidence for the alternative ( $BF_{10}$ = 3.93); however, there was anecdotal evidence *against* both a mixing cost ( $BF_{01}$ = 1.31; d = 0.24 [−0.02, 0.50]) and a switch cost ( $BF_{01}$ = 2.07; d = 0.17 [−0.05, 0.40]).

#### Experiment 1b

Analysis of proportion accuracy (Figure 2a) across Sequence showed no evidence for the alternative ( $BF_{10}$ = 1.69). Analysis of RT (Figure 2b) across Sequence showed extreme evidence for the alternative ( $BF_{10}$ = 276.59); there was moderate evidence for a mixing *benefit* (cf., cost; $BF_{10}$ = 5.87; d = 0.12 [0.03, 0.21])) and extreme evidence for a switch cost ( $BF_{10}$ = 5,858.72; d = 0.18 [0.11, 0.25])). For the signal detection analysis, analysis of sensitivity (Figure 2c) across Sequence showed moderate evidence for the null ( $BF_{10}$ = 0.24). Analysis of response bias (Figure 2d) across Sequence showed extreme evidence for the alternative ( $BF_{10}$ = 132,702.80); pairwise comparisons showed extreme evidence for a mixing cost ( $BF_{10}$ = 111.19; d = 0.72 [0.32, 1.13])), but no evidence for a switch cost ( $BF_{10}$ = 1.17; d = 0.34 [0.01, 0.67])).

### Discussion

Experiment 1 provides evidence for a cost to visual short-term memory performance on dimension switch trials in mixed-block conditions where participants need to switch to encoding a new feature dimension (cf., feature dimension repetitions). There were no consistent effects of mixing cost. For mixed-block performance, there was a switch cost for accuracy for bivalent stimuli (Experiment 1a) but not for univalent stimuli (Experiment 1b), a pattern which also manifested in sensitivity ( $d^{\prime }$ ; i.e. a switch cost in Experiment 1a, but no evidence for a cost in Experiment 1b). In addition, although there was a numerical switch cost for response bias in both experiments, the evidence for this was weak; however, for both experiments, the pattern was the same in that response bias was highest for pure blocks, intermediate for mixed repetition trials, and lowest for switch trials. The lack of evidence for switch costs on this measure, therefore, may be a power issue. There was a clear switch cost in response time in both experiments; this effect is consistent with task switching research, but as response time is not a typical outcome measure in visual short-term memory experiments, we leave its interpretation to the General Discussion.

These results together provide initial evidence that feature dimension switching disrupts visual short-term memory performance, and that this is to some extent moderated by the feature load of each object, with costs to accuracy and sensitivity when the feature load was higher (and stimuli were bivalent) in Experiment 1a in comparison to Experiment 1b. However, there were some inconsistencies in results across experiments for different outcome measures, so in Experiment 2, we replicate and extend these findings.

## Experiment 2

We conducted four experiments (Experiments 2a–2d) providing a conceptual replication of Experiment 1 utilising similar stimuli to that of Markov et al. (2019). These authors were interested in whether features from different dimensions are stored independently, and—if so—whether this independence is supported regardless of whether the features belong to the same or different objects. They presented participants with feature dimensions of colour and orientation, and were asked to memorise both features (cf., our experiments, where only one feature dimension is cued). After a delay period, participant was probed to recall the feature value of one of the memoranda and reproduce it in on a continuous scale (e.g. colour/orientation wheel). The main manipulation relevant to the current paper was whether the feature dimensions belonged to the same or different objects.

**Figure 3.**
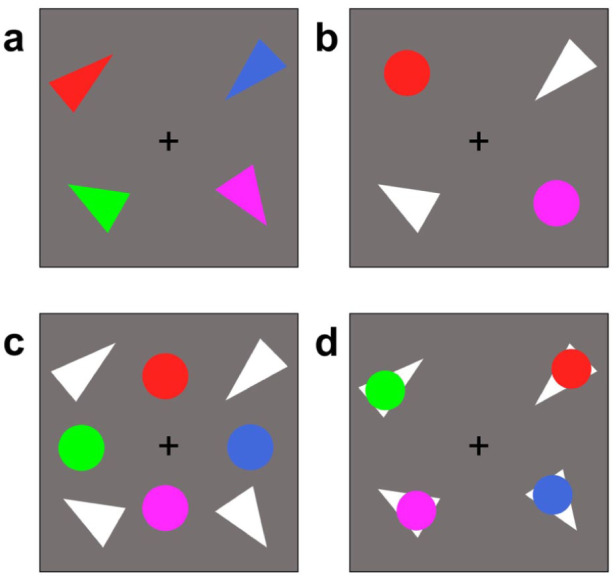
Example stimuli from Experiments 2a–2d.

The stimuli used in the current experiments are shown in Figure 3 corresponding to Experiments 2a–2d. In Experiment 2a, stimuli were coloured, oriented isosceles triangles with both feature dimensions (colour and orientation) integrated into the same object (similar to Experiment 1a). There were four objects, and the feature load of each object was two. In Experiment 2b, stimuli were distinct unidimensional objects comprising coloured circles and oriented white triangles, with feature dimensions distributed across objects (similar to Experiment 1b). There were four objects, and the feature load of each object was one. These two experiments together provided conceptual replications of Experiments 1a and 1b. Experiment 2c extended Experiment 2b by increasing the number of unidimensional objects to eight—four coloured circles and four oriented triangles. This increases the overall number of objects (from two to four), and the effective set size of each feature dimension (from two to four). Stimuli in Experiment 2d were the same as in Experiment 2c (coloured circles and oriented triangles), but were spatially overlapping such that each oriented triangle had a coloured circle placed on top. This arrangement may lead participants to encode these items as unified bivalent objects due to perceptual grouping principles. If so, the overall number of objects is four, and the effective set size of each feature dimension is two; if not, the overall number of objects is eight, and the effective set size of each feature dimension is one.

This overall configuration of stimuli across conditions allows us to ask a number of questions. The primary question is whether we replicate the switch cost in accuracy and signal detection measures (and with lesser interest, response time). If so, such a switch cost may reflect difficulty in resource allocation on switch trials relative to repetition trials. Our design further allows us to ask whether this resource allocation is moderated by (a) feature integration vs. overlap (e.g. Experiment 2a vs. 2d); total set size and integration vs. distribution of features (e.g. Experiment 2a vs. 2b); selective increases in effective set size (Experiment 2b vs. 2c); manipulation of integrated vs. spatially separated features (Experiment 2a vs. 2c); and spatial separation vs. spatial overlap of features (Experiment 2c vs. 2d).

We also made other modifications from Experiment 1 in order to enhance the sensitivity to detect effects of dimension switching on vSTM performance. First, we removed the pure blocks, so participants were only exposed (after practice) to mixed-block trials where repetitions and switches were equally likely. This allowed for an increase in the number of trials. In addition, we utilised a sequential Bayes factor design (Schönbrodt & Wagenmakers, 2018) to better determine the sample size of each experiment.

### Method

#### Participants

Sample size for each experiment was determined by sequential Bayes factor analysis (Schönbrodt & Wagenmakers, 2018). We established that our minimum sample size would be 20, and our maximum (due to time and resources) would be 70. Once the minimum sample size had been reached, we conducted a Bayesian *t*-test on proportion accuracy comparing repetition and switch performance. If the $BF_{10}$ was >10 or <0.1—indicating strong evidence in support of the alternative or null hypothesis, respectively—data collection was terminated. If neither threshold was reached, data collection continued with the test being performed again after every 5 participants until either of the BF thresholds or the maximum sample size was reached.

The total number of recruited participants was 47, 73, 72, and 75 for Experiments 2a–2d, respectively. As in Experiment 1, participants were removed if their performance was not statistically better than chance. This led to a final sample size for analysis of 45, 70, 70, and 70 for Experiments 2a–2d. Participants were recruited through a combination of the participant panel run by the School of Psychology at Keele University and Prolific, with unique participants in each experiment. All participants completed the study online. Participants were aged between 18 and 60 (inclusive), and self-reported normal or corrected-to-normal visual acuity and colour vision. Prolific recruitment was limited to the United Kingdom and United States. Participants recruited via Prolific were paid a small fee, and participants recruited via the Psychology participant panel received partial course credit.

#### Materials

The task was again a change detection task; however, different stimuli were used (see Figure 3). In Experiment 2a, the stimuli consisted of four bivalent (coloured and oriented) isosceles triangles. In Experiment 2b, four univalent stimuli were presented: two of the stimuli were coloured circles, and two were white triangles. The stimuli of Experiments 2a and 2b therefore mimic the stimuli and effective set sizes used in Experiments 1a and 1b (respectively). In Experiment 2c, eight univalent stimuli were presented: four coloured circles, and four white triangles. In Experiment 2d, as in 2c, eight univalent stimuli were presented; however, unlike Experiment 2c, in Experiment 2d, the stimuli were spatially overlapping such that each white triangle had a coloured circle on top. Feature values for colour and orientation were selected as in Experiment 1; in Experiments featuring the white univalent triangles, participants were informed that white would never be a colour-dimension feature value. Dimension cues were identical to Experiment 1, as were the stimulus frames and fixation crosses. The experiment was programmed and delivered using Gorilla (Anwyl-Irvine et al., 2020).

#### Procedure

The procedure was similar to Experiment 1 with the exception that only mixed blocks were used. Therefore, participants made a change detection judgement in blocks of trials wherein the relevant feature dimension could repeat (e.g. colour–colour) or switch (e.g. colour–orientation) from the previous trial. A total of 40 practice trials were presented (10 colour only trials; 10 orientation only trials; 20 mixed trials) followed by 400 mixed trials separated into blocks of 50. There was a self-paced rest screen at the end of each block.

### Results

The analytical approach was different from that of Experiment 1. Here, data across Experiments 2a–2d were analysed in a single model. Specifically, we calculated Bayes factors for factorial designs as described by Rouder et al. (2017), with the factors *Sequence* (repetition vs. switch, within-subjects) and *Experiment* (2a–2d, between-subjects). To assess the evidence in favour of each factor (plus the interaction), we first constructed a full model which included all predictors (one predictor for each factor plus one for their interaction). Then, separate models were constructed that selectively omitted one predictor at a time. The evidence for each predictor is the Bayes factor for the omitted model compared to the Bayes factor for the full model. Specifically, the Bayes factor for a particular predictor is calculated as $BF_{\mathrm{Predictor}}=\frac{BF_{\mathrm{Omitted}}}{BF_{\mathrm{Full}}}$ . Bayes factors lower than one is considered evidence in favour of the full model and—by extension—evidence in favour of inclusion of that particular predictor; Bayes factors larger than one are considered evidence in favour of the omitted model, and therefore evidence against inclusion of that particular predictor.

**Figure 4.**
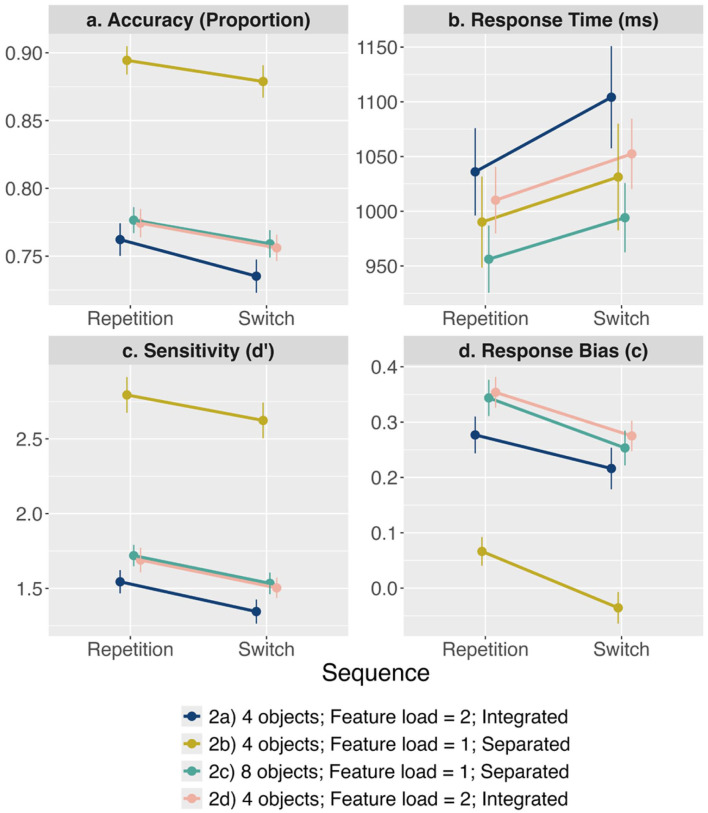
The results from Experiment 2a–2d. Each panel represents a different dependent variable (a) Accuracy, (b) Response Time, (c) Sensitivity, and (d) Response Bias.

The results are shown in Figure 4. For the accuracy analysis, there was extreme evidence for both the main effect of Sequence ( $BF_{\mathrm{Sequence}}=4.31\times 10^{-7}$ ) and the main effect of Experiment ( $BF_{\mathrm{Experiment}}=2.78\times 10^{-18}$ ). However, there was very strong evidence against the interaction ( $BF_{\mathrm{Interaction}}=30.58$ ). Effect sizes for switch costs for Experiments 2a–2d, respectively, were d = 0.33 [0.14, 0.52], d = 0.16 [0.06, 0.26], d = 0.21 [0.06, 0.36], and d = 0.21 [0.06, 0.37]. The main effect of experiment was followed up with a series of Bayesian *t*-tests for each pairwise experiment comparison. The Bayes factors for these comparisons can be seen in Table 1. There was extreme evidence for a difference in accuracy between Experiment 2a and 2b, between Experiments 2b and 2c, and between Experiments 2b and 2d.

**Table 1.** Bayes Factors ( $BF_{10}$ ) From Bayesian *t*-Tests for Between-Experiment Comparisons (a–d) for Each Dependent Variable in Experiment 2.

Comparison	Accuracy	RT	*d*’	c
a vs. b	2.83×10^7^	0.29	$7.57\times 10^{9}$	$3.93\times 10^{5}$
a vs. c	0.43	0.91	0.76	0.36
a vs. d	0.34	0.26	0.49	0.65
b vs. c	$5.32\times 10^{10}$	0.22	$1.62\times 10^{10}$	$1.95\times 10^{9}$
b vs. d	$8.14\times 10^{10}$	0.19	$3.50\times 10^{10}$	$8.00\times 10^{11}$
c vs. d	0.18	0.38	0.19	0.20

*Note.* RT = response time; *d*’ = sensitivity; c = response bias; Experiment 2a = 4 objects, feature load of 2, integrated features; Experiment 2b = 4 objects, feature load of 1, separated features; Experiment 2c = 8 objects, feature load of 1, separated features; Experiment 2d = 4 objects, feature load of 2, integrated features.

For response time, there was extreme evidence for the main effect of Sequence ( $BF_{\mathrm{Sequence}}=1.51\times 10^{-12}$ ), but anecdotal evidence against the main effect of Experiment ( $BF_{\mathrm{Experiment}}=2.59$ ). There was strong evidence against the interaction ( $BF_{\mathrm{Interaction}}=16.30$ ). Effect sizes for switch costs for Experiments 2a–2d, respectively were d = 0.21 [0.11, 0.32], d = 0.09 [0.03, 0.15], d = 0.14 [0.09, 0.20], and d = 0.16 [0.09, 0.22].

For sensitivity, there was extreme evidence for the main effect of Sequence ( $BF_{\mathrm{Sequence}}=3.82\times 10^{-9}$ ) and extreme evidence for the main effect of Experiment ( $BF_{\mathrm{Experiment}}=8.00\times 10^{-21}$ ). However, there was very strong evidence against the interaction ( $BF_{\mathrm{Interaction}}=52.91$ ). Effect sizes for switch costs for Experiments 2a–2d, respectively, were d = 0.37 [0.18, 0.57], d = 0.17 [0.05, 0.29], d = 0.31 [0.17, 0.44], and d = 0.28 [0.13, 0.44]. The Bayes factors for the between-experiment comparisons showed extreme evidence for a difference in accuracy between Experiment 2a and 2b, between Experiments 2b and 2c, and between Experiments 2b and 2d.

For response bias, there was extreme evidence for the main effect of Sequence ( $BF_{\mathrm{Sequence}}=1.22\times 10^{-7}$ ) and extreme evidence for the main effect of Experiment ( $BF_{\mathrm{Experiment}}=2.00\times 10^{-13}$ ). However, there was very strong evidence against the interaction ( $BF_{\mathrm{Interaction}}=33.51$ ). Effect sizes for switch costs for Experiments 2a–2d, respectively, were d = 0.25 [0.05, 0.45], d = 0.45 [0.16, 0.73], d = 0.34 [0.15, 0.53], and d = 0.34 [0.17, 0.51]. The Bayes factors for the between-experiment comparisons showed extreme evidence for a difference in accuracy between Experiment 2a and 2b, between Experiments 2b and 2c, and between Experiments 2b and 2d.

### Discussion

The results of Experiment 2 were clear: Switching feature dimension incurred a cost to all measures of visual short-term memory performance. Importantly, all dependent variables demonstrated switch costs that did not interact with the between-experiment manipulations of set size, feature load, or feature overlap.

In addition, the results offer insight into a potential explanation of the reduction of accuracy and sensitivity with dimension switching. For most experiments and conditions, response bias ( c ) was above zero, which indicates a general tendency for participants to report “no-change.” However, c was lower on switch trials, reflecting a shift toward a more liberal bias—a lower tendency to report “no-change.” One interpretation is that on switch trials, the now-irrelevant (but previously *relevant*) feature dimension continues to receive vSTM resources. This may occur due to either proactive interference caused by persisting activation of the previously relevant feature dimension (Allport et al., 1994), or because reconfiguration to the new feature dimension is incomplete (Rogers & Monsell, 1995) and therefore the irrelevant feature dimension receives some resources. As there was always a change present in the irrelevant feature dimension, the proactive interference and/or incomplete reconfiguration may make this change more salient on switch trials, increasing the likelihood of a “change” response even when the relevant dimension remains unchanged. We return to this interpretation in the General Discussion.

## Experiment 3

**Figure 5.**
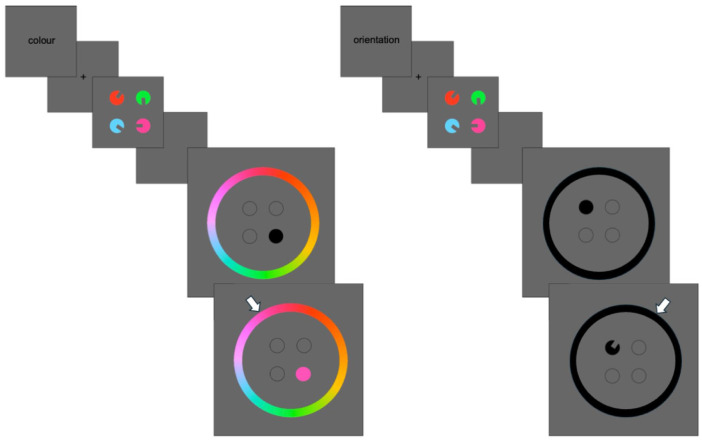
Example trials from Experiment 3. The left shows a colour trial where participants are cued to memorise the colour of the stimuli. At the probe screen, one of the stimulus locations is indicated (filled black circle), and a colour wheel is presented. Participants move their mouse on the colour wheel (indicated by the white arrow, not visible to participants), which changes the colour of the probed location to match the hue on the colour wheel. The right shows an example location trial.

The purpose of Experiment 3 was to generalise the findings from the first two experiments to the delayed estimation paradigm (Prinzmetal et al., 1998; Wilken & Ma, 2004). Recall that in this paradigm, participants are presented with memoranda to encode similarly to experiments presented thus far; however, at test, participants are probed to the location of one of the memoranda, and are asked to recall and reproduce the probed object’s feature value on a continuous wheel. The novel extension in Experiment 3 is to embed this delayed estimation task within a task switching paradigm. Similar to Experiments 1 and 2, participants are cued on each trial to encode either the colour or orientation of four bivalent circular shapes with a rectangular segment removed to indicate orientation. At test, participants are probed to recall the task-relevant feature value of a probed object; if the task is colour, participants reproduce the feature value on a colour wheel, and if the task is orientation, participants reproduce the orientation on an orientation wheel (see Figure 5). Memory precision can therefore be inferred from the magnitude of response error (calculated as the angular deviation between the true feature value and the participant’s response), with higher memory precision leading to smaller response error.

As well as assessing the generalisability of the findings from Experiments 1 and 2, employing the delayed estimation task allowed us to apply a formal mathematical model of visual short-term memory in order to identify a mechanistic explanation of the source of the switch cost so far observed. Specifically, we applied the three-component mixture model of Bays et al. (2009). This model formalises the proposal that response errors arise due to (a) noisy internal representations of the true feature values, (b) incorrectly retrieving the feature value of one of the non-probed objects (a form of binding error), and (c) random guessing when memory fails.

The three-component model is defined as

(3) $$\begin{array}{c} p\left(\hat{\theta }\right)=p_{t}\phi_{\kappa }\left(\hat{\theta }-\theta \right)+p_{g}\frac{1}{2\pi }+p_{nt}\frac{1}{n}\underset{i\in nt}{\sum }\phi_{\kappa }\left(\hat{\theta }-\theta_{i}^{*}\right) \end{array}$$

where $\hat{\theta }$ is the participant’s response (in radians), θ is the target’s (i.e. the probed object’s) feature value on the relevant cued dimension (in radians), and $\theta_{i}^{*}$ is the feature value of the non-target stimulus i in the set of all non-targets nt (i.e. $\theta_{1}^{*}$ , $\theta_{2}^{*}$ , and $\theta_{3}^{*}$ ). $p_{nt}$ and $p_{g}$ are the probabilities of making a non-target and guess response, respectively, and the probability of a target response, $p_{t}$ , is therefore by $1-p_{nt}-p_{g}$ . $\phi_{\kappa }$ represents the probability density of the von Mises distribution centred on zero with concentration parameter κ .

The key parameters are therefore κ , reflecting memory precision (with higher values represent higher precision), $p_{nt}$ , reflecting probability of responding to one of the non-target’s feature values, and $p_{g}$ , representing the probability of guessing. Fitting the model to our paradigm will allow us to establish whether dimension switching impacts memory precision ( κ ), feature binding (i.e. $p_{nt}$ ), or guessing ( $p_{g}$ ).

### Method

#### Participants

A total of 50 participants completed Experiment 3 in an in-person study. All participants were aged between 18 and 60 and reported normal or corrected-to-normal visual acuity and colour vision. Participants were recruited via the participant panel run by the School of Psychology at Keele University and were awarded course credit or paid a small fee for taking part.

Sample size was again determined by a sequential Bayes factor analysis, with a minimum sample size of 20 and a maximum sample size of 70. When the minimum sample size was reached, the participant exclusion criterion was checked (see “Results” section). Specifically, we removed participants whose probability of guessing (as determined by the $p_{g}$ parameter in the mixture model) was >0.5; this was because simulations have shown that estimates of other mixture model parameters become unstable when guessing rates are high (Grange & Moore, 2022). Once we had at least 20 participants who passed the exclusion check, we conducted a Bayesian paired-samples *t*-test on the mean absolute error of participant responses (repetition vs. switch). If the Bayes factor for this test ( $BF_{10}$ ) was >10 (strong support for the alternative) or lower than 0.1 (strong support for the null), data collection was terminated. If neither threshold was reached, another five participants were recruited, and the process was repeated. In total, 7 participants were removed, and the threshold was reached after 43 participants.

#### Materials

The experiment was programmed and delivered using PsychoPy (Peirce et al., 2019). The experiment was run on a 24.5 inch monitor (ASUS ROG Swift^TM^ PG258Q) at a viewing distance of 52 cm. which was held constant through use of a chin rest. Stimuli were similar to Experiment 1a in that the memory display comprised of four coloured circles with rectangular segments removed to provide orientation (see Figure 5). Colours were selected from a colour wheel situated within the CIE L-a-b colour space (radius = 60; L = 90; a = 20; b = 38). Each stimulus had a radius of 30 pixels (px.). The background was light grey (RGB: 170, 171, 171), and the cues and fixation crosses were presented in black. The cues were “colour” and “orientation” for the colour and orientation dimensions, respectively. On the test display, four empty black circles were presented to represent the location of each of the memoranda’s locations from the memory display. One of these black circles were filled black to indicate which location was relevant for recall (i.e. the “probe”). These location templates were surrounded by a circular colour wheel (on colour trials) or a circular wheel that was completely black (on orientation trials). These wheels had a radius of 250 px. and width of 35 px.

#### Procedure

Participants were first provided a demonstration of the task requirements by the software, which walked through three trials each for colour and orientation dimensions. After this, participants completed a practice session of 20 trials (10 colour-only trials, followed by 10 orientation-only trials). Participants then completed the main experimental blocks, which consisted of 8 blocks of 50 trials, with self-paced rest at the end of each block.

Each trial began with the presentation of a cue indicating which feature dimension was relevant on the current trial. The relevant cue was randomly selected and was presented for 500 ms. The cue was then replaced by a fixation cross for 500 ms, after which time the four stimuli of the memory display were presented for 200 ms. The colour of each stimulus was randomly selected as locations on the colour wheel (i.e. from 1 to 360 degrees) with the constraint that there be at least 40 degrees separation between each selected colour so as to minimise stimulus confusability. Similarly, orientation of each stimulus was randomly selected (from 1 to 360 degrees) with the same 40-degree minimum separation constraint. The display then went blank for a retention interval of 1,000 ms, after which the probe screen was presented, which remained on screen until a response from the participant was registered. The probe display consisted of the four templated locations, as well as a colour wheel (on colour trials) or black wheel (on location trials; see Figure 5). One of the four memoranda was probed for recall based on location; for example, if the bottom-right stimulus location was probed, the participant was required to recall the feature value of the relevant dimension for the stimulus in that position on the memory display (i.e. recall the colour or orientation, depending on the current trial’s cue).

Participants responded by moving the mouse cursor to the location on the wheel which they believed best matched the feature value of the probed stimulus. When the mouse was clicked over the wheel, the probed stimulus would take on the feature value under the cursor; for example, on colour trials, if the participant moved their cursor towards a pinkish hue, the probe stimulus would take on the same hue (see Figure 5). If the participant moved the mouse to a different location whilst holding the click down, the probe would dynamically take on the feature value of what colour the mouse was over. The participant finalised their response by pressing the space bar; responses were not time-limited so participants were free to move their cursor to different locations on the wheel prior to selecting their final response. However, participants were instructed to respond as quickly and accurately as possible. On each trial, the response location of the finalised response (in degrees) was recorded, as well as response time. Once a response was provided, the display was removed for 500 ms, after which time a fixation cross appeared for 500 ms, and then the cue for the next trial was presented.

As the relevant feature dimension was randomly selected, across participants there were roughly equal number of repetition trials (*M* = 192, *SD* = 9.98, min. = 170, max. = 211) and switch trials (*M* = 188, *SD* = 9.76, min. = 171, max. = 211). Parameter recovery simulations have shown that around 200 trials provide very accurate parameter estimation of the three-component model (Grange & Moore, 2022).

### Results

Prior to analysis, participant exclusion checks were conducted. First, trials in which the response time was shorter than 150 ms or longer than 2.5 standard deviations above the mean per participant per cell of the design were removed. Then, the three-component mixture model was fitted to individual participant data (see the “Mixture Modelling” section for details), and participants who had a probability of guessing (as estimated via the $p_{g}$ parameter of the model) over 0.5 were removed.

#### Behavioural Analysis

**Figure 6.**
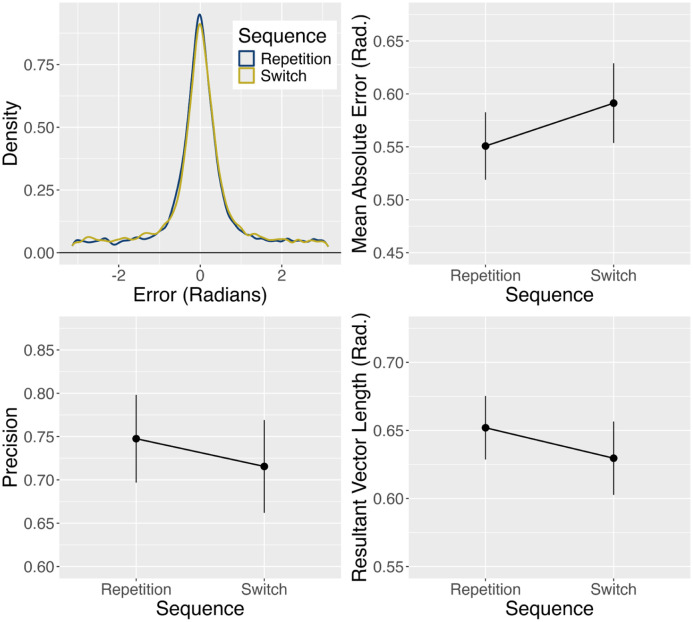
Model-free summary statistics of response error from Experiment 3. Error bars denote ± one standard error around the mean. *Note.* Rad. = Radians.

There was extreme evidence that response times were shorter for dimension repetition trials (*M* = 2,559, *SE* = 110) than for dimension switch trials (*M* = 2,638, *SE* = 115), $BF_{10}$ = 143.87, d = 0.10 [0.05, 0.15]. For the response error analysis, responses were converted to radians, and a measure of response error was calculated by subtracting the participant’s response (in radians) from the true value of the probed target (in radians) on each trial; by this metric, perfect recall of the target feature value would result in a response error of zero. The density distribution of response errors across all participants is shown in Figure 6 (top-left). As can be seen, performance was good with the peak of the error distribution centred at zero; the non-zero density in the tails of the distribution represents imprecise memory recall and/or guessing. Although the error distributions are similar for repetition and switch trials, repetition has higher density around zero indicative of higher precision in recall on dimension repetition trials.

Response error was summarised using the model-free metrics of precision, mean absolute error, and resultant vector length (calculated via the mixture R package, Grange & Moore, 2023): Mean absolute error is the circular mean of the (absolute) response error, with higher values reflecting larger response error; precision is the reciprocal of the standard deviation of responses (for circular data) accounting for values expected by chance^4^; and resultant vector length measures response error variability, with values close to one indicating little error variability and responses close to zero indicating response errors spread uniformly across the circle.

These metrics are summarised in Figure 6 and were analysed via Bayesian paired samples *t*-tests (one for each metric). Analysis showed a strong effect of sequence for mean absolute error ( $BF_{10}$ = 13.88; d = 0.16 [0.06, 0.26]), with larger error on switch trials compared to repetition trials. There was moderate evidence *against* an effect for precision ( $BF_{10}$ = 0.324; d = 0.09 [−0.06, 0.25]), and no evidence for an effect for resultant vector length ( $BF_{10}$ = 1.68; d = 0.13 [0.01, 0.24]). In sum, these results suggest dimension switching had a strong effect on the response error of participants.

#### Mixture Modelling

The three-component model of Bays et al. (2009) was fitted to individual participant data per sequencing condition using the R package *mixtur* (Grange & Moore, 2023). The model fitting provided—for each participant and for each level of dimension sequence—three model parameters: κ , the concentration parameter of the von Mises distribution (with higher values reflecting more precise memory representations); $p_{n}t$ , the probability of basing the response the recall of one of the non-target values; and $p_{g}$ , the probability of guessing. $p_{t}$ —the probability of basing the response on recall of the target value—is then calculated as $1-p_{nt}-p_{g}$ . Parameters were estimated via maximum likelihood, and a wide range of starting parameters were explored in the fitting routine (for further details, see Grange & Moore, 2023).^5^

**Figure 7.**
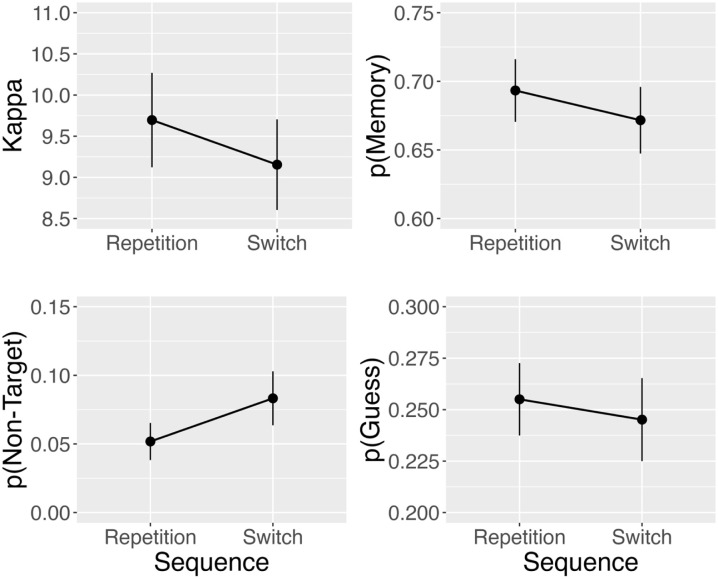
Mean parameter estimates from the three-component mixture model. Error bars denote ± one standard error around the mean.

Mean parameter values can be seen in Figure 7. Analysis of κ provided moderate support *against* an effect of sequence ( $BF_{10}$ = 0.23; d = 0.15 [−0.20, 0.49]). There was no effect of sequence on $p_{t}$ ( $BF_{10}$ = 0.85; d = 0.14 [−0.01, 0.29]), but there was a moderate effect of sequence on $p_{n}$ ( $BF_{10}$ = 3.09; d = 0.25 [0.05, 0.45]). As can be seen in Figure 7 (bottom-left), the probability of responding to a non-target feature value was increased on switch trials compared to repetition trials. There was moderate evidence *against* an effect of sequence on $p_{g}$ ( $BF_{10}$ = 0.20).

### Discussion

Experiment 3 generalised the change detection findings from Experiments 1 and 2 to a delayed estimation paradigm. Results showed a strong effect of dimension switching on mean absolute error, replicating and extending the findings from Experiments 1 and 2 of impaired visual short-term memory performance on dimension switch trials relative to dimension repetition trials. The use of a delayed estimation task allowed us to apply the three-component model of Bays et al. (2009) to explore potential mechanistic explanations of the source of the dimension switch cost to vSTM. This model decomposes response errors in the delayed estimation task into (a) memory imprecision due to noisy internal representations of the true feature value, (b) erroneous recall of a non-probed object’s feature value, and (c) random guessing when memory fails.

Results from the modelling showed a selective—albeit modest—increase in the probability of retrieving a non-target feature value on dimension switch trials relative to dimension repetition trials. No other parameter was impacted by dimension switching, suggesting that dimension switching did not affect the quality of memory representation (i.e. κ ), and therefore speaks against a purely resource-based explanation of the dimension switch cost. Instead, the findings suggest dimension switching impacted the binding of feature values to location which—when location is later used as a recall cue at the probe stage—leads to greater confusion in selecting the correct item in memory. This results in an increase in non-target responses without reducing the quality of feature memory itself, and is consistent with the idea that dimension switching in the delayed estimation task impairs object selection rather than memory fidelity.

## General Discussion

The goal of the present study was to investigate how changes in task demands within dynamic environments impacts the allocation of limited visual short-term memory (vSTM) resources to task-relevant features. Previous work has established that resource allocation is responsible for the fidelity of feature representations in memory, with features receiving more resources being represented in memory with greater precision (Bays & Husain, 2008; Bays et al., 2009, 2024; Ma et al., 2014). Therefore, these limited resources must be accurately applied to task-relevant features so that task-irrelevant features do not consume limited resources. We embedded two classic vSTM paradigms within a task switching paradigm where the relevant feature dimension could repeat (e.g. colour–colour) or switch (e.g. colour–orientation) from that of the previous trial. On dimension switch trials, some degree of cognitive control is required to update the relevant task set (Grange, 2025) so that the limited resources are applied to the correct (i.e. goal-relevant) feature dimension.

We found a clear negative impact of dimension switching on vSTM performance that replicated and generalised across both change detection (Experiments 1 and 2) and delayed estimation (Experiment 3) paradigms, as well as across different stimulus types (Experiments 2a–2d). This impairment was evidenced by reduced accuracy and sensitivity (i.e. $d^{\prime }$ ; Experiments 1a and 2a–2d), as well as a reduction in response bias on switch trials (Experiments 1a and Experiments 2a–2d) during change detection; although response bias was above zero in all conditions (reflecting a bias to report “no-change”), on switch trials bias was lower reflecting a higher likelihood to report “change.” We suggest that this shift in bias was influenced by the constant change that occurred on the irrelevant feature dimension, and as such suggests that on switch trials the irrelevant feature dimension was influencing responses. We also observed increased response error during delayed estimation (Experiment 3); fitting the three-component mixture model (Bays et al., 2009) to this data showed no switch-related change in memory precision ( κ ) or guessing ( $p_{g}$ ), but a selective increase in non-target responses ( $p_{nt}$ ). The observed dimension switch costs were therefore robust, generalising across task type (e.g. Experiments 1 and 2 vs. Experiment 3) and stimulus type (e.g. Experiments 2a–2d).

### Theoretical Interpretation

Our main expectation in this study—as outlined in the introduction—was that dimension switching would lead to weaker memory representations due to suboptimal encoding of memoranda caused by a reduction in—or a misallocation of—memory resources (Bays & Husain, 2008; Bays et al., 2009). The reduction in sensitivity ( $d^{\prime }$ ) in Experiments 1a and 2a–2d is compatible with this view; we also observed a robust reduction in response bias ( c ) on switch trials, reflecting a higher likelihood of responding “change,” likely driven by the constant change in the irrelevant (but recently relevant) feature dimension, which might be caused by resources being misapplied on switch trials. However, the data from Experiment 3 speak against a view of reduced memory quality: Applying the three-component mixture model of Bays et al. (2009) to delayed estimation response errors in Experiment 3 showed no impact of dimension switching on memory precision (estimated via the κ parameter) or guessing ( $p_{g}$ ). Instead, we found a selective increase in feature–location binding errors (reflected by an increase in the $p_{nt}$ parameter), which suggests switching impacted the quality of feature–location binding (Oberauer & Lin, 2017; Schneegans & Bays, 2017).

As the task demands are quite different between change detection and delayed estimation (Lin & Oberauer, 2022), it is possible that the negative impact of dimension switching on vSTM performance may arise from different mechanisms in each paradigm. However, we propose that the collective findings can be explained by binding errors induced by incomplete attentional reconfiguration of the relevant task set and/or persisting activation of the irrelevant (but previously relevant) task set on switch trials. Binding is a core mechanism in many formal models of vSTM (Oberauer & Lin, 2017; Schneegans & Bays, 2017, 2019). For example, in the conjunctive coding model of Schneegans and Bays (2017), multi-feature objects are encoded and maintained via separate neural population codes that bind each single feature dimension to location (e.g. there is one conjunctive code binding colour and location, and another conjunctive code binding orientation and location). On switch trials, incomplete reconfiguration of the relevant feature dimension and/or persisting activation of the previous (but now irrelevant) feature dimension may induce noise into the binding process.

This framework naturally explains the observed increase in non-target responses in the delayed estimation task in Experiment 3 (as measured by the $p_{nt}$ ) parameter. At test, location (e.g. top-left) serves as a cue to recall the feature value on the relevant feature dimension (e.g. colour) of the object at the cued location. In the conjunctive coding framework, this is achieved by decoding the population activity corresponding to all feature–location bindings; the object whose location code most closely matches the cued location is selected, and its value on the relevant dimension is reported (Bays, 2016; McMaster et al., 2022; Oberauer & Lin, 2017; Souza et al., 2014). If dimension switching impairs feature–location binding, there is an increased probability that the location cue activates a non-target object, leading to an increased probability of reporting non-target feature values (consistent with increased $p_{nt}$ ).

The framework can also explain the pattern of results found for change detection (i.e. reduction in $d^{\prime }$ and c ). During encoding on switch trials, the system may not have fully reconfigured to prioritise the currently relevant feature dimension and/or there may be persisting activation of the previously relevant (but now irrelevant) feature dimension. The net effect is a degradation of the encoding of the relevant feature–location bindings and/or greater prioritisation of the irrelevant feature–location bindings. At test, participants compare the test display to their memory to ascertain whether a change has occurred. This involves decoding the stored feature–location activation and assessing whether any object has changed in the relevant dimension. If switching impairs the precision or accessibility of the relevant bindings, change detection will be impaired (reflected in poorer accuracy and lower $d^{\prime }$ , as observed). In addition, interference from the irrelevant feature–location bindings (which always changed in our experiments) may increase decisional uncertainty and bias participants to reporting a change, as reflected by lower response bias c , as observed.

The conjunctive coding framework may therefore help to provide a unified account of the varied findings across experiments, suggesting a common underlying mechanism in which incomplete reconfiguration of the relevant feature dimension and/or persisting activation of the irrelevant feature dimension leads to degradation of conjunctive representations. We acknowledge, however, that this framework provides a natural explanation for the delayed estimation data and that its application to the change detection data remains tentative and requires further research. Such work should include formal modelling of both change detection and delayed estimation data (Lin & Oberauer, 2022; e.g. using the interference model framework, Oberauer & Lin, 2017), which will be valuable to test whether a single mechanism can explain dimension switch costs across vSTM paradigms.

#### Object-Based vs. Feature-Based Representation

Although the current study was not designed to address whether vSTM representations are object-based or feature-based, our finding of a robust dimension switch cost contributes to this debate. Specifically, we suggest that our findings are compatible with feature-based representation in vSTM. If memoranda were represented at the object level (D. Lee & Chun, 2001; Luck & Vogel, 1997; Ngiam et al., 2024; Ramaty & Luria, 2025; Sone et al., 2021; Vogel et al., 2001), then both feature dimensions (colour and orientation) of each stimulus should be encoded together regardless of the cued dimension. As a consequence, the object-based account makes a prediction of no switch cost: Once integrated objects are stored, all of their features should be equally available, and switching the relevant dimension should not impair performance. In contrast, the feature-based account (Bays et al., 2011, 2024; Brady et al., 2011, 2024; Brown et al., 2021; Fougnie et al., 2010; Li et al., 2022; Markov et al., 2019, 2021; Marshall & Bays, 2013; Shin & Ma, 2016, 2017) does predict a switch cost because on switch trials the system must prioritise resource allocation to the currently cued feature dimension which can be error-prone due to either incomplete reconfiguration of the currently relevant task set or persisting activation from the previous—and now irrelevant—task set.

Our findings are therefore hard to reconcile with object-based representation in vSTM and instead favour feature-based representation. However, it is possible that the representation could be object-based and switch costs emerge at the decision or response stage rather than during encoding and/or maintenance.

### Future Research and Limitations

Our main theoretical explanation requires further empirical work supplemented by fitting of *explanatory* models, which—in contrast to *measurement* models that aim to quantify latent psychological processes—provide mechanistic explanations for behaviour (for a discussion of measurement vs. explanatory models, see Oberauer & Lin, 2017). One obvious candidate is fitting the extended neural population model of Schneegans and Bays (2017; which extends the neural population model of Bays, 2014), which would allow a more direct assessment of our proposal that dimension switching impacts feature–location binding in conjunctive coding. Lin and Oberauer (2022) have provided a framework in which explanatory models of vSTM can be applied to a variation of the change detection paradigm by extending the interference model of Oberauer and Lin (2017), which would facilitate our efforts to integrate our findings between change detection (which we could not model given our design^6^) and delayed estimation (which we could model). This is an important next step in the research programme.

Another avenue for future research is to fit variants of the three-component model to our delayed estimation data (Experiment 3). For example, the model we fitted (Bays et al., 2009) assumes fixed precision of memory representations across memoranda and across trials [i.e. fixed κ ]. Research has shown that models with variable precision—across trials as well across items within a trial—generally provide better fits to delayed estimation data (Fougnie et al., 2012; van den Berg et al., 2012). Indeed, the seminal modelling study by van den Berg et al. (2014)—whereby the authors compared 32 models of vSTM that varied across three factors, including whether precision is equal or variable across items and trials—showed that classes of models with variable precision fit delayed estimation data better than classes of models with fixed precision. It is important, therefore, in future research to fit such variable precision models to the data from Experiment 3 to establish whether our conclusions hold.

Future modelling work could also benefit from taking into account response time. In our studies, we consistently found robust switch effects in response time, which is a common outcome variable in studies of task switching. In the task switching literature, response time is the primary dependent variable, and switch costs are thought to reflect the delay caused by the time it takes to reconfigure task set parameters (Rogers & Monsell, 1995; see Grange, 2025), the time it takes to resolve proactive interference from the previously relevant task (Allport et al., 1994), or both (Meiran, 2000; Meiran et al., 2000). We refrained from interpreting these switch costs as response time is not a commonly used dependent variable in vSTM studies. However, they could provide an important constraint on theoretical explanations of our effects. Recent extensions of the diffusion framework—which is a prominent modelling architecture in speeded decision-making tasks (Voss et al., 2013)—to data from the delayed estimation paradigm uses the so-called circular diffusion model (Smith, 2016; Smith et al., 2020) which provides a theory of both speed and precision of responses. Such a framework would allow us to incorporate effects of dimension switching on response time into our theoretical explanations, and remains an important avenue for future research.

The current study’s broader contribution is to the potential for theoretical integration across domains in cognitive science (visual short-term memory and cognitive control during task switching) to provide complementary perspectives. For example, working memory gating models (Chatham & Badre, 2015; Chatham et al., 2014; O’Reilly & Frank, 2006) propose that cortico-striatal structures provide gating mechanisms that control what information enters working memory, and of that information what is then allowed to influence ongoing behaviour. In our study, the observed switch cost could be explained by a failure to effectively gate the currently relevant feature dimension allowing the intrusion of the irrelevant feature dimension to vSTM. In addition, the dual mechanism theory of cognitive control framework (Braver, 2012) proposes that control processes act in a proactive or a reactive manner. The observed switch costs in the current study, therefore, may reflect a failure to engage proactive control (leading to incomplete reconfiguration of the currently relevant dimension, DeJong, 2000), thereby necessitating late reactive control. In the task switching literature, there is a rich history of distinguishing between proactive control (i.e. task-set reconfiguration theories, Rogers & Monsell, 1995) and reactive control (i.e. overcoming the effects of task-set inertia, Allport et al., 1994). The current paradigm, therefore, has the potential to speak to these broader concepts via empirical and theoretical integration.

In terms of limitations, it is important to note that there was some inconsistency in results in the change detection experiments. Specifically, whilst Experiment 1a found switch costs in accuracy and sensitivity, this was not the case for Experiment 1b. However, there were very strong switch cost effects for these outcome variables in Experiment 2. In addition, there was no clear statistical effect (although there was a numerical effect) of bias in Experiment 1, but this was strong in Experiment 2. We view Experiment 2 as the better experiment—and hence weight our interpretations more from this experiment—for several reasons. First, Experiment 1 was primarily exploratory as it was the first in this research programme. We therefore had fewer participants, and as such, the observed inconsistencies (both across Experiments 1a and 1b, as well as across Experiments 1 and 2) could be a power issue. Experiment 2 had much better power—both within each sub-experiment individually, but also collectively as data were pooled across sub-experiments in the analysis. Contributing to lower power was the fact that we had three levels of dimension sequence in Experiment 1 (pure repetition, mixed repetition, and switch), meaning each condition had fewer trial numbers (Experiment 1 had 150 pure block trials, and 300 mixed block trials; each sub-experiment of Experiment 2 had 400 mixed block trials, and there were 4 sub-experiments). It is likely, therefore, that design issues contributed to the lack of consistency in some of the findings. It is important to note, though, that in Experiment 2, there were strong switch costs in all outcome variables, and these switch costs did not vary across subexperiment.

Although the switch cost was robust across the vast majority of experiments (e.g. Experiment 1a, Experiments 2a–2d, and Experiment 3), the magnitude of the effects observed is rather small. Design aspects could have reduced the magnitude of these effects. For example, in the change detection experiments, the test screen on the current trial appeared 3,700 ms after the response from the previous trial (taking into account inter-trial intervals, cuing intervals, fixation time, encoding time, etc.). In the delayed estimation task, whilst the time between the response to the previous trial and the onset of the test screen on the current trial was much shorter (2,200 ms), it is still much longer than typically seen in task-switching studies. Whilst we made these design choices to be consistent with timings typically found in vSTM studies, research in task switching has consistently shown that increasing the inter-trial intervals can have strong effects on the reduction of the magnitude of switch costs (Kiesel et al., 2010; Meiran, 2000; Rogers & Monsell, 1995; Vandierendonck et al., 2010). Thus, the effect size of the observed switch costs is likely reduced by these design factors, and future work should look to find ways to reduce the inter-trial intervals without compromising the ability to compare findings to the broader vSTM literature.

We also recruited a wide age range of participants (18–60 years). Although age was not a primary variable of interest in the current study, it remains possible that age-related changes to cognitive control and visual short-term memory could have impacted our results. Future research should either restrict the age range further or include participant age as an additional predictor in the analysis.

Another limitation is that—like many studies of vSTM—the stimuli we employed are rather basic and abstracted away from the complexities of real-world items. Recent research has been interested in adapting studies of visual memory to incorporate more complex, meaningful stimuli (Brady et al., 2019, 2024), and research has generally shown that memory is enhanced for such realistic stimuli (Chung et al., 2024; Markov et al., 2021; Sahar et al., 2024). The extent to which our findings generalise to such contexts remains an important avenue for future work.

## Conclusion

In conclusion, our study is, to our knowledge, the first to explore the impact of dimension switching on visual short-term memory performance. Collectively, the results provide evidence for a cost to performance on dimension switch trials. We suggest our findings reflect a limitation of dynamic prioritisation of relevant feature dimensions that impacts feature–location binding, and therefore that visual short-term memory is sensitive to attentional control failures, not just capacity limits.

## Supplemental Material

sj-pdf-1-qjp-10.1177_17470218251404415 – Supplemental material for The Impact of Dimension Switching on Visual Short-Term Memory Supplemental material, sj-pdf-1-qjp-10.1177_17470218251404415 for The Impact of Dimension Switching on Visual Short-Term Memory by Stuart B. Moore and James A. Grange in Quarterly Journal of Experimental Psychology

## Notes

1. We included a wide range of allowable ages so as to maximise data collection. Unfortunately, beyond this, we did not formally record the age of our participants as this was not of interest to our main research questions. We have since gone back to the demographics data that Prolific hold for our participants. For Experiment 1a, demographic data from 17 participants were no longer available on Prolific (likely due to account closure); for the remainder, the mean age was 37.5 years (*SD* = 10.85). For Experiment 1b, demographic data were unavailable from 10 participants, and the median age for the remainder was 37 years (*SD* = 11.57).

2. In two pilot studies, a change only occurred on the relevant feature dimension on change trials (i.e. values on the irrelevant feature dimension remained unchanged from memory display to test display). However, in such situations, we realised that participants could perform successfully without needing to attend to the cued feature dimension. Instead, participants could monitor the display for *any* change (regardless of dimension). Such *whole display monitoring* would negate any need for dimension switching as the task-set would remain the same throughout the experiment: monitor for change irrespective of which feature dimension is relevant.

3. Specifically, we used R (Version 4.5.1; R Core Team, 2023) and the R-packages *BayesFactor* (Version 0.9.12.4.7; Morey & Rouder, 2024), *bmm* (Version 1.2.0; Frischkorn & Popov, 2023), *coda* (Version 0.19.4.1; Plummer et al., 2006), *dplyr* (Version 1.1.4; Wickham, François, et al., 2023), *forcats* (Version 1.0.0; Wickham, 2023), *ggplot2* (Version 3.5.2; Wickham, 2016), *lubridate* (Version 1.9.4; Grolemund & Wickham, 2011), *Matrix* (Version 1.7.3; Bates et al., 2023), *mixtur* (Version 1.2.2; Grange & Moore, 2023), *papaja* (Version 0.1.3; Aust & Barth, 2023), *patchwork* (Version 1.3.1; Pedersen, 2024), *plyr* (Wickham, 2011; Version 1.8.9; Wickham, François, et al., 2023), *PrettyCols* (Version 1.1.0; Rennie, 2024), *purrr* (Version 1.1.0; Wickham & Henry, 2023), *readr* (Version 2.1.5; Wickham, Hester, & Bryan, 2023), *sdtt* (Version 0.2.0; Moore, 2024), *stringr* (Version 1.5.1; Wickham, 2022), *tibble* (Version 3.3.0; Müller & Wickham, 2023), *tidyr* (Version 1.3.1; Wickham, Vaughan, & Girlich, 2023), *tidyverse* (Version 2.0.0; Wickham et al., 2019), *tinylabels* (Version 0.2.5; Barth, 2023) and *trimr* (Version 1.1.1; Grange, 2022).

4. If participants perform completely at random, response errors will be distributed uniformly on the circle. If this is not accounted for, then precision is non-zero even when participants respond truly randomly. Calculation of precision, therefore, corrects for this by subtracting the expected precision under a uniform distribution from the observed precision of response errors so that true guessing results in a precision of zero.

5. The quality of the resulting model fit was assessed in three ways. First, in the Supplemental Appendix, we provide visualisations of observed and model-predicted response error distributions for each participant and each sequence. As can be seen, the model predictions closely captured participants’ response error distributions. Second, we calculated pseudo- $R^{2}$ values by calculating for each participant and each sequence the log-likelihood under a uniform-only null model. The pseudo- $R^{2}$ is then $1-\frac{LL_{\mathrm{Model}}}{LL_{\mathrm{Null}}}$ . Across participants and sequence, the mean pseudo- $R^{2}$ was .372, indicating very good model fit. Third, we calculated the Akaike information criterion for the null model and the three-component model and took the difference ( Δ ). The results showed that the three-component model was decisively favoured over the uniform null model for every participant (mean Δ = 254, *SD* = 112).

6. The extension of the interference model to change detection paradigms by Lin and Oberauer (2022) was developed for the single-probe change detection where memory is tested at a single location, and participants respond as to whether that single item has changed from the memory array. Our experiments presented a full-array change detection task in which all locations are probed at test (although a change, if present, only occurred in a single location). The full-array version requires multiple comparisons between memory and the test display (one for each item), making the comparison process difficult to model.
