# Supplementary material for: The Impact of Dimension Switching on Visual Short-Term Memory
Source: Q J Exp Psychol (Hove). 2025 Nov 28;79(8):2020–40. doi: 10.1177/17470218251404415 (PMC13400800; doi:10.1177/17470218251404415)
Supplement: sj-pdf-1-qjp-10.1177_17470218251404415 – Supplemental material for The Impact of Dimension Switching on Visual Short-Term Memory [file sj-pdf-1-qjp-10.1177_17470218251404415.pdf]

Appendix — Assessment of Model Fit Quality

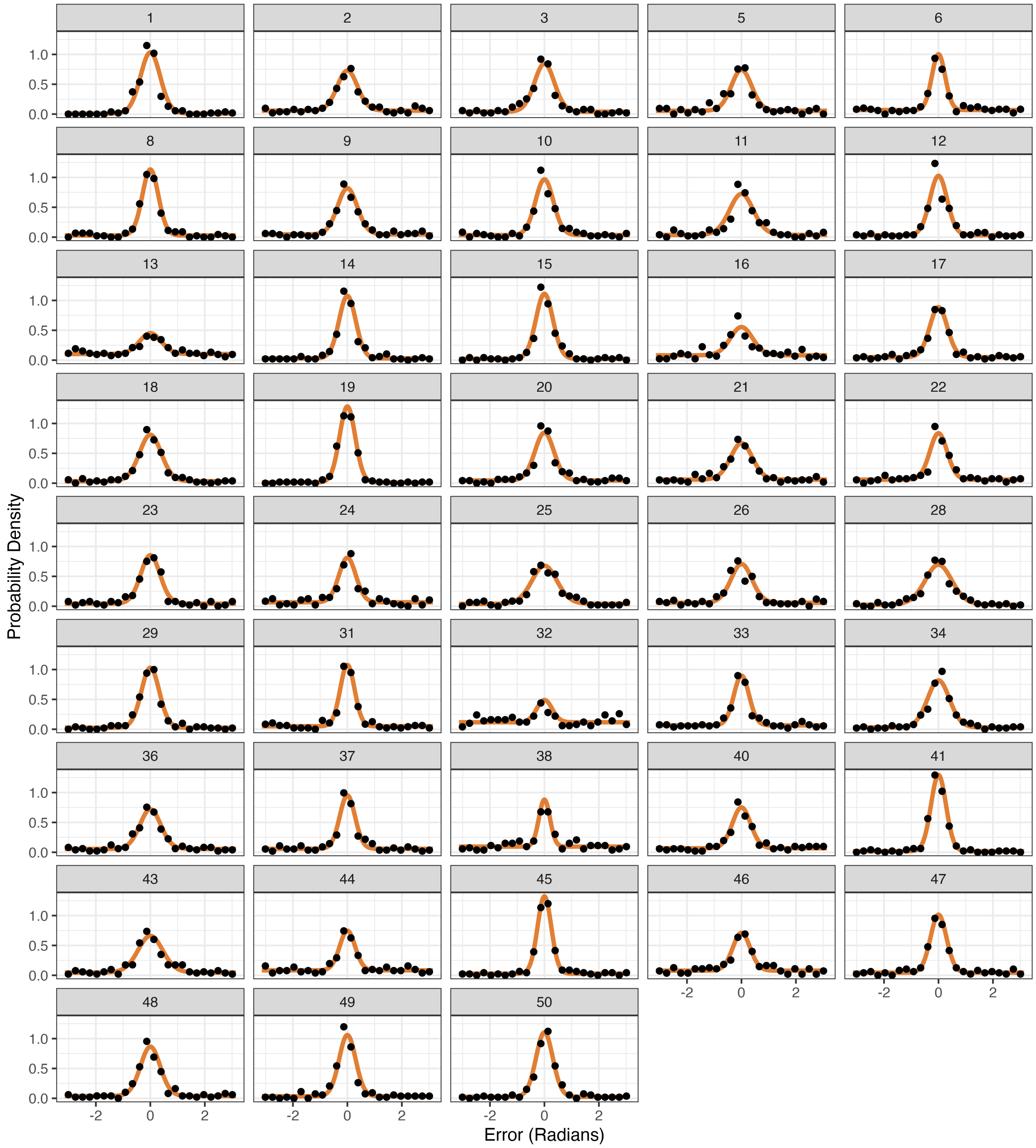

Figure 8. Observed (black dots) and predicted (orange line) response error distributions for each individual participant (one participant per panel) for dimension repetition trials in Experiment 3.

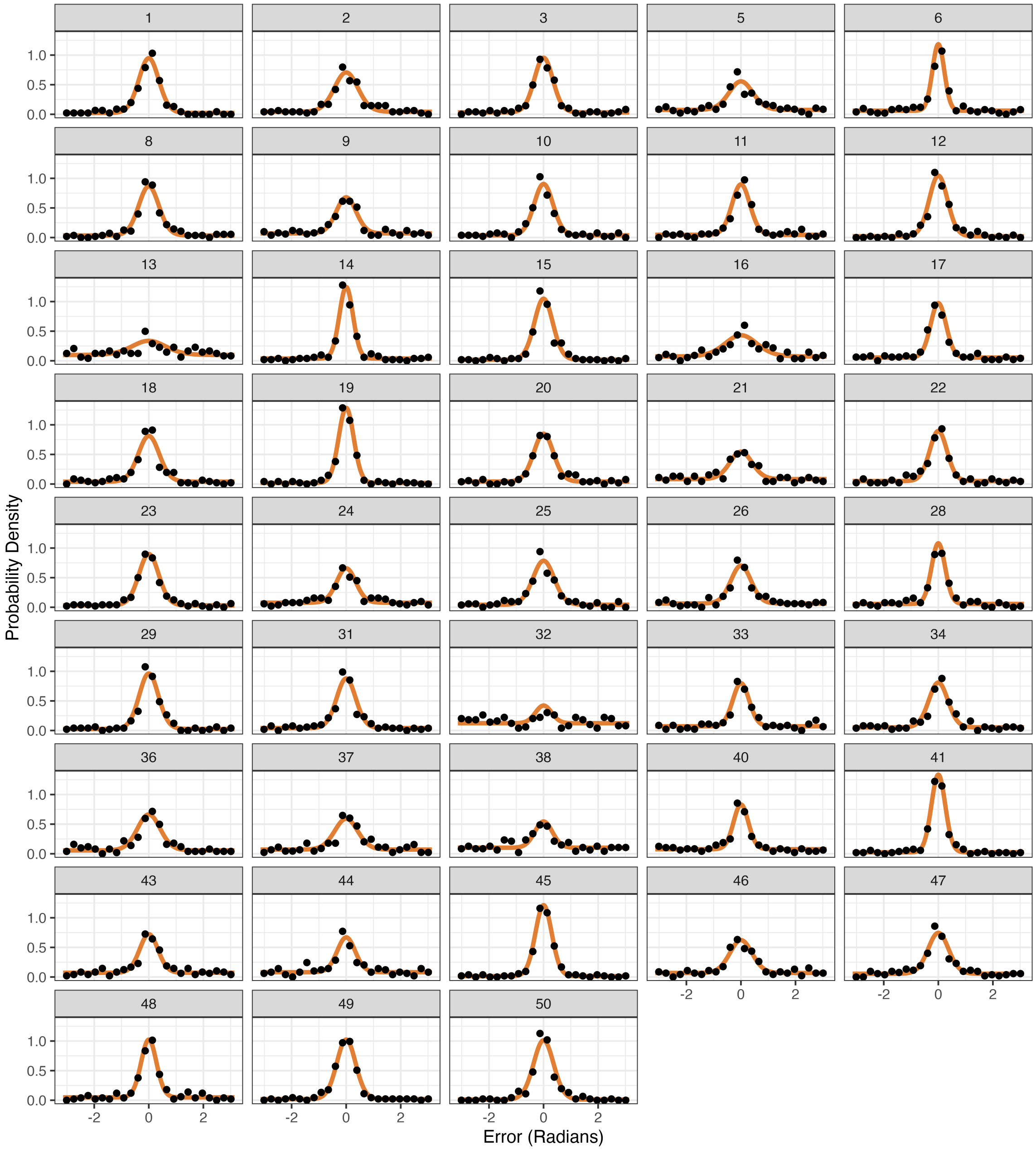

Figure 9. Observed (black dots) and predicted (orange line) response error distributions for each individual participant (one participant per panel) for dimension switch trials in Experiment 3.
